# Off the Beaten Path: A Thyroglossal Duct Cyst in the Sublingual Space of a 31‐Year‐Old Male—A Case Report

**DOI:** 10.1155/crot/8832072

**Published:** 2026-04-09

**Authors:** Michael C. Oca, Farhoud Faraji, Omonigho Aisagbonhi, Andrew M. Vahabzadeh-Hagh

**Affiliations:** ^1^ Department of Otolaryngology Head and Neck Surgery, University of California San Diego, La Jolla, California, USA, ucsd.edu; ^2^ Department of Pathology, University of California San Diego, La Jolla, California, USA, ucsd.edu

## Abstract

Thyroglossal duct cysts (TGDCs) are among the most common congenital anomalies of the neck, typically presenting as painless midline masses inferior to the hyoid bone. Distinguishing TGDCs from other cystic neck lesions, such as ranulas or dermoid cysts, can be challenging, particularly when they arise in uncommon locations such as the sublingual space. Here, we report an atypical presentation of a TGDC in the sublingual space of a 31‐year‐old Hispanic/Latino White male. The patient, with no significant medical history, presented to the emergency department at the University of California San Diego Health (Hillcrest Medical Center, San Diego, California, United States), in June 2023 after sustaining blunt facial trauma. Imaging revealed an incidental cystic lesion in the right paramedian floor of the mouth. Magnetic resonance imaging (MRI) confirmed a circumscribed cystic mass (21 × 21 × 20 mm) within the sublingual space. Imaging characteristics, including the lesion’s close approximation to the hyoid bone and absence of restricted diffusion, were most compatible with a TGDC. MRI also confirmed a normal thyroid gland and no cervical lymphadenopathy. Surgical excision of the cyst, including a segment of the hyoid bone, was performed. Histopathological examination confirmed the diagnosis of a TGDC with respiratory and squamous epithelial lining. At the 1‐week postoperative follow‐up, flexible laryngoscopy showed no complications, with normal vocal fold function. TGDCs in the sublingual space are extremely rare, with only nine previously reported cases in the English literature. This report underscores the significance of recognizing atypical presentations of TGDCs, particularly in rare locations such as the sublingual space, expanding the understanding of their diverse clinical manifestations.

## 1. Introduction

Congenital anomalies of the thyroglossal duct represent one of the most common etiologies for pediatric neck masses, second only to lymphadenopathy among cervical lesions in children [1, 2]. The thyroglossal duct cyst (TGDC) accounts for approximately 70% of congenital midline neck anomalies and has a reported prevalence of up to 7% in the general population, affecting males and females with equal frequency [2–6]. TGDCs typically present as painless midline neck masses inferior to the hyoid bone that characteristically elevate with deglutition and tongue protrusion [3, 4]. While most TGDCs are located in the thyrohyoid (60.9%), suprahyoid (24.1%), suprasternal (12.9%), or intralingual (2.1%) regions, rare presentations outside the typical thyroglossal tract pose unique diagnostic challenges [3, 4]. Sublingual TGDCs are particularly difficult to distinguish from other cystic lesions of the floor of the mouth, including ranulas, dermoid cysts, epidermoid cysts, and cystic hygromas [1, 3]. To date, only nine cases of TGDCs arising in the oral floor or sublingual space have been reported in the English literature, with most occurring in pediatric patients aged 6–14 years [3]. Here, we describe an atypical presentation of a TGDC arising in the sublingual space of an adult male, incidentally discovered on imaging following facial trauma. The purpose of this report is to detail the diagnostic workup, imaging findings, surgical management, and histopathological confirmation of this rare entity, and to reinforce the importance of including TGDCs in the differential diagnosis of sublingual cystic lesions. Patient consent was obtained.

## 2. Case Presentation

In June 2023, a 31‐year‐old White Hispanic male with no pertinent medical history was admitted to the emergency department following a blunt trauma to the face with a baseball (University of California San Diego Health, San Diego, California, USA). The acute onset of symptoms included two episodes of emesis and blurred vision in the right eye. Computed tomography (CT) imaging of the facial bones demonstrated an acute right orbital floor fracture. In addition, the CT scan uncovered a coincidental benign‐appearing cystic lesion, measuring 18 mm, in the right paramedian floor of mouth deep to the mylohyoid muscle.

**FIGURE 1:**
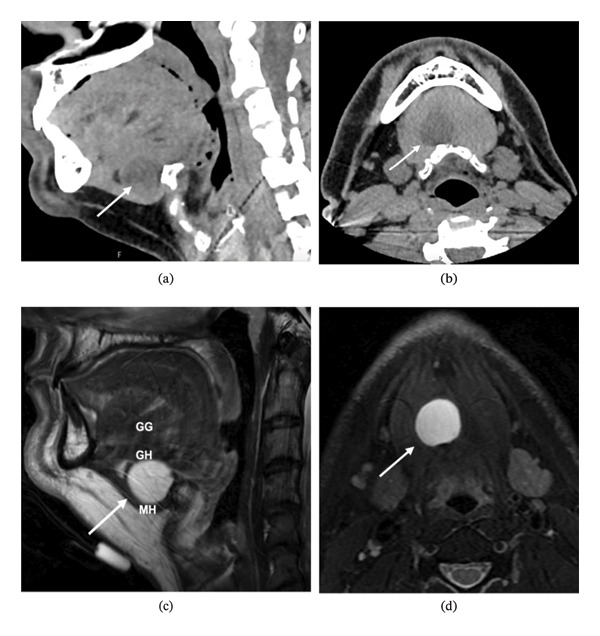
(a) Sagittal and (b) axial contrast‐enhanced computed tomography demonstrating a cystic lesion (arrows) in the sublingual space, deep to the mylohyoid muscle. (c) Sagittal and (d) axial T2 weighted magnetic resonance imaging of the same lesion (arrows); genioglossus (GG), geniohyoid (GH), and mylohyoid (MH).

Subsequent noncontrast magnetic resonance imaging (MRI) of the neck soft tissue confirmed the presence of a circumscribed cystic mass (21 × 21 × 20 mm) centered within the sublingual space, deep to the mylohyoid muscle. The lesion appeared homogeneous, slightly hyperintense on T2 weighted imaging, and marginally hyperintense relative to muscle on T1 weighted imaging, with no internal enhancement but a thin rim of peripheral enhancement. No restricted diffusion was identified. The mass was positioned immediately anterior to the hyoid bone, slightly right of midline, with minimal underlying hyperostotic change as previously identified on CT scan (Figure 1). The MRI also confirmed a normal thyroid gland and the absence of cervical lymphadenopathy. The absence of restricted diffusion made dermoid cyst much less likely, and the findings were most compatible with a TGDC.

**FIGURE 2:**
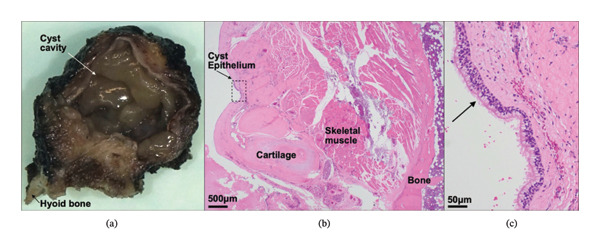
(a) Gross photograph of the thyroglossal duct cyst showing cyst cavity (arrow) and fragment of attached hyoid bone (arrowhead). Hematoxylin and eosin–stained sections at (b) 2 × magnification showing cyst epithelium (arrow) with attached cartilage, skeletal muscle, and bone (dashed box indicates region shown in (c)), and (c) 20 × magnification showing respiratory epithelium lining the cyst (arrow).

Intraoperatively, the tumor was identified as a well‐circumscribed cyst situated within the genioglossus/geniohyoid muscle complex. The lesion was excised *en bloc*, along with the median segment of the hyoid bone. Histopathological examination demonstrated a respiratory and squamous epithelium‐lined cyst attached to skeletal muscle and hyoid bone, confirming the diagnosis of a TGDC. Postoperative follow‐up was conducted at one day and 1 week after surgery. At the 1‐week visit, the patient reported expected pain with neck motion and swallowing but was otherwise progressing well. Flexible laryngoscopy showed that the vocal folds adducted and abducted normally and symmetrically with a patent glottic airway. The patient was advised to continue light activity, and return precautions were provided. No further follow‐up visits occurred beyond this date (Figure 2).

## 3. Discussion

TGDCs are observed in approximately 7% of the population affecting male and female individuals equally [2, 5, 7]. They are most commonly located in four distinct areas: thyrohyoid (60.9%), suprahyoid (24.1%), suprasternal (12.9%), and intralingual (2.1%) [3, 4, 8]. The origin of these cysts can be traced back to the 4th week of embryological development when the thyroid anlage, an invagination of endodermal cells arising from the ventral floor of the pharynx, accompanies the descent of the heart and great vessels caudally toward the loose prepharyngeal connective tissue [9]. By the 7th week of gestation, the epithelial trace left behind, known as the thyroglossal tract, reaches its final position in the inferior pretracheal neck [10]. Consequently, these cell remnants may persist anywhere along this developmental pathway, extending from the foramen cecum area to the suprasternal notch. Notably, the thyroid gland’s typical migration route bypasses the sublingual area, making TGDCs of this area an extremely rare occurrence [3, 11]. In fact, there have only been nine previously reported cases in the English literature of TGDCs developing in the oral floor, with most instances occurring in individuals 6–14 years of age [3].

Typically, TGDC manifests as a painless midline mass adjacent to the hyoid bone [3, 12], with elevation of the mass upon deglutition and tongue protrusion [3, 12]. However, in this atypical presentation of a TGDC in the sublingual space, its association to swallow and tongue motion may be more challenging to evaluate. In addition, unlike most TGDCs which arise posterior to the cervical strap muscles, this cyst’s unusual location deep to the mylohyoid required a more complex dissection. Several additional factors warrant consideration when evaluating TGDCs in atypical locations. Preoperative evaluation of suspected TGDCs should include assessment of the thyroid gland, as thyroid hemiagenesis has been reported in association with thyroglossal duct anomalies, and excision without confirming the presence of orthotopic thyroid tissue risks iatrogenic hypothyroidism [13, 14]. In our patient, MRI confirmed a normal, intact thyroid gland. Similarly, cervical lymphadenopathy may accompany TGDCs in the setting of infection or malignant transformation, broadening the differential to include metastatic disease or infected branchial cleft cysts [1, 4]. No lymphadenopathy was identified in our patient, consistent with the incidental, noninflamed nature of the cyst. The differential diagnosis of sublingual cystic lesions includes ranulas, dermoid cysts, epidermoid cysts, and cystic hygromas [1]. In this case, the absence of restricted diffusion on MRI argued against a dermoid cyst, which typically demonstrates diffusion restriction due to keratinous debris. The close approximation of the lesion to the hyoid bone and its paramedian position favored a TGDC over a simple ranula.

The tissue diagnosis of TGDC depends on the presence of respiratory pseudostratified ciliated columnar and squamous epithelium, often associated with a thyroglossal tract or thyroid follicles within the surrounding tissue [12]. In the presented case, though no definite thyroid follicles were identified, the diagnosis was confirmed by the respiratory and squamous epithelium‐lined cyst attached to skeletal muscle and the hyoid bone (Figure 2). These findings are consistent with previous studies that have demonstrated that TGDCs occurring above the hyoid bone are most likely to consist of pseudostratified epithelium [15], further solidifying the relationship between the epithelial lining of TGDCs and their location at the time of diagnosis.

## 4. Conclusion

This case report highlights an atypical presentation of a TGDC in the sublingual space, expanding the current understanding of the diverse manifestations of these cysts. Advanced imaging modalities, particularly MRI with diffusion‐weighted sequences, play a valuable role in distinguishing sublingual TGDCs from other cystic lesions of the floor of the mouth, including dermoid cysts and ranulas. Preoperative confirmation of a normal, intact thyroid gland is essential in any patient undergoing excision of a suspected TGDC, given the rare but clinically significant association with thyroid hemiagenesis. Recognizing TGDCs in unusual locations is important for accurate diagnosis and appropriate management, and clinicians should maintain a broad differential when evaluating cystic lesions of the sublingual space.

## Author Contributions

Michael C. Oca: manuscript preparation, radiographic imaging preparation, writing, and editing.

Farhoud Faraji: radiographic imaging preparation and manuscript preparation, writing, editing.

Omonigho Aisagbonhi: pathology analysis, images, and descriptive text.

Andrew M. Vahabzadeh‐Hagh: radiographic imaging preparation and manuscript preparation, writing, and editing.

## Funding

This research received no specific grant from any funding agency in the public, commercial, or not‐for‐profit sectors.

## Disclosure

No funding was allocated for this manuscript as it was performed as part of the employment from the University of California San Diego Health Sciences, Department of Surgery, Division of Otolaryngology.

## Consent

Patient consent was obtained.

## Conflicts of Interest

The authors declare no conflicts of interest.

## Data Availability

The data that support the findings of this study are available from the corresponding author upon reasonable request.
